# On the Probabilistic Deployment of Smart Grid Networks in TV White Space

**DOI:** 10.3390/s16050671

**Published:** 2016-05-10

**Authors:** Angela Sara Cacciapuoti, Marcello Caleffi, Luigi Paura

**Affiliations:** 1 Department of Electrical Engineering and Information Technologies, University of Naples Federico II, Naples 80138, Italy; marcello.caleffi@unina.it (M.C.); luigi.paura@unina.it (L.P.); 2 Multimedia Communications Laboratory, CNIT, Naples 80126, Italy

**Keywords:** white space, smart grid, cognitive radio, sensor network

## Abstract

To accommodate the rapidly increasing demand for wireless broadband communications in Smart Grid (SG) networks, research efforts are currently ongoing to enable the SG networks to utilize the TV spectrum according to the Cognitive Radio paradigm. To this aim, in this letter, we develop an analytical framework for the optimal deployment of multiple closely-located SG Neighborhood Area Networks (NANs) concurrently using the same TV spectrum. The objective is to derive the optimal values for both the number of NANs and their coverage. More specifically, regarding the number of NANs, we derive the optimal closed-form expression, *i.e.*, the closed-form expression that assures the deployment of the maximum number of NANs in the considered region satisfying a given collision constraint on the transmissions of the NANs. Regarding the NAN coverage, we derive the optimal closed-form expression, *i.e.*, the closed-form expression of the NAN transmission range that assures the maximum coverage of each NAN in the considered region satisfying the given collision constraint. All the theoretical results are derived by adopting a stochastic approach. Finally, numerical results validate the theoretical analysis.

## 1. Introduction

To accommodate the rapidly increasing demand for wireless broadband communications in Smart Grid (SG) scenarios, research efforts [1] are currently ongoing to enable the SG Networks (SGNs) to utilize the channels of the TV spectrum temporarily unused by the licensed users, referred to as incumbents, according to the Cognitive Radio paradigm [2,3,4,5,6]. The vacant channels are known as TV White Space (TVWS) channels.

SG communications over TVWS channels are conditioned by regulators on the ability of the SGNs to avoid harmful interference toward incumbents. To this aim, the existing rulings [7] obviate the spectrum sensing as the mechanism for the SGNs to determine the TVWS availability. Instead, they require the SGNs to periodically access to a geolocated database, referred to as White Space DataBase (WSDB) [8,9,10], for acquiring the list of TVWS channels.

**Figure 1:**
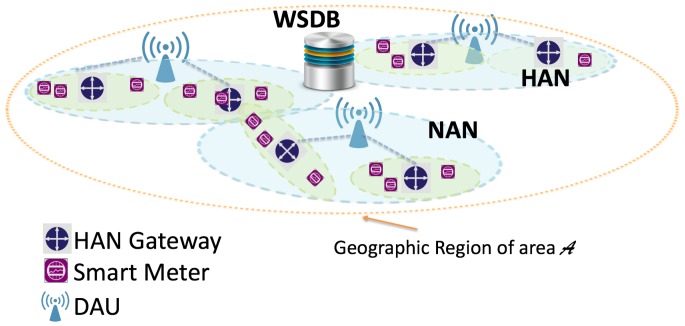
Smart Grid Scenario: Multiple Closely-Located Neighborhood Area Networks (NANs).

Let us consider the typical SG scenario shown in Figure 1, where several smart meters connected to a gateway constitute a Home Area Network (HAN), and multiple gateways connected to a Data Aggregate Unit (DAU) constitute a Neighborhood Area Network (NAN) [1,11,12] (please note that the typical wireless technologies and the corresponding values of the coverage ranges for HANs and NANs are reported in [12], along with further details on their communication features). The gateway is responsible for transmitting the meter data periodically collected within its HAN to the DAU via a TVWS channel, once declared available by the WSDB. However, experimental studies have shown that the number of TVWS channels is significantly limited in urban areas [13]. Hence, it is likely that multiple closely-located NANs are authorized to use the same TVWS channel and their transmissions may collide, leading so to a performance degradation. Moreover, since so far there are no regulatory requirements for the coexistence among NANs operating in TVWS spectrum, such a performance degradation can be severe. The described scenario of multiple closely-located NANs sharing the same TVWS channel has been considered for the first time in [14], where the sensing duration that maximizes the achievable data rate at a given NAN has been determined.

Differently from [14], in this letter, we develop an analytical framework for the optimal deployment of multiple closely-located NANs using the same TVWS channel with the objective to derive the optimal values for both the number of NANs and their coverage. More specifically, regarding the number of NANs, we derive the optimal closed-form expression, *i.e.*, the closed-form expression that allows to evaluate the maximum number of NANs in the considered network region satisfying a given collision constraint on the transmissions of NANs.

Regarding the NAN coverage, we derive the optimal closed-form expression, *i.e.*, the closed-form expression of the NAN transmission range that assures the maximum coverage of each NAN in the considered network region satisfying the given collision constraint.

All theoretical results are derived by adopting a stochastic approach and by considering the specificity of the NAN traffic demands. Finally, numerical results validate the theoretical analysis.

## 2. System Model

In a geographic region of area A, *K* NANs are deployed. The HAN gateways of each NAN are responsible for transmitting the meter data periodically collected within their HANs to the DAU via the same TVWS channel declared available by the WSDB, as depicted in Figure 1. We denote with $G_{i}$ the number of HAN gateways belonging to the *i*-th NAN and with $R_{\text{TX}}^{i}$ their transmission range. The traffic of the *i*-th NAN is modeled thought the parameters $P_{\text{a}}^{i}$ and $P_{\text{in}}^{i}\overset{▵}{=}1-P_{\text{a}}^{i}$ denoting the probabilities of an arbitrary HAN gateway belonging to the *i*-th NAN being active (*i.e.*, having meter data collected within its HAN to transmit to the DAU) and inactive, respectively.

## 3. Optimal Deployment Analysis

At a certain time instant *t*, the transmissions of two arbitrary HAN gateways, say $H_{\ell }^{i}$ and $H_{m}^{j}$ belonging to the *i*-th and *j*-th NANs, respectively, collide if and only if the HAN gateways are both active and their distance $d_{H_{\ell }^{i}H_{m}^{j}}\left(t\right)$ (denoted in the following as $d_{ij}\left(t\right)$ for the sake of simplicity) is smaller than the distance threshold constituted by the maximum between their transmission ranges, *i.e.*, $d_{ij}\left(t\right)\leq \max\left(R_{\text{TX}}^{i},R_{\text{TX}}^{j}\right)$. Hence, it results that the transmissions of a certain HAN gateway belonging to the *i*-th NAN, say $H_{\ell }^{i}$, collide with the transmissions of the *j*-th NAN if it exists al least one active HAN gateway belonging to the *j*-th NAN within the distance threshold. We denote the probability of this event as $P_{\text{C}}^{H_{\ell }^{i},j}$. As a consequence, there is no collision between the transmissions of NANs *i* and *j* if none of the active HANs belonging to NAN *i* is within the distance threshold of any of the active HANs belonging to NAN *j*. We denote the probability of this event as ${\bar{P}}_{\text{C}}^{ij}$. In the following, by accounting for these definitions, with some algebraic manipulations, we derive the expressions of $P_{\text{C}}^{H_{\ell }^{i},j}$ and $P_{\text{C}}^{ij}\overset{▵}{=}1-{\bar{P}}_{\text{C}}^{ij}$. Specifically:

(1) $$P_{\text{C}}^{H_{\ell }^{i},j}\overset{▵}{=}1-{\bar{P}}_{\text{C}}^{H_{\ell }^{i},j}=1-\left[P_{\text{in}}^{i}+P_{\text{a}}^{i}{\left(1-P_{\text{a}}^{j}P_{d_{ij}}\right)}^{G_{j}}\right]$$

where $P_{d_{ij}}$ denotes the probability of the event $\left\{d_{ij}\left(t\right)\leq \max\left(R_{\text{TX}}^{i},R_{\text{TX}}^{j}\right)\right\}$. By accounting for Equation (1) and the definition of $P_{\text{C}}^{ij}$, in the reasonable hypothesis of independence of the HAN transmissions, it results

(2) $$\begin{array}{c} P_{C}^{ij}\overset{▵}{=}1-{\bar{P}}_{C}^{ij}=1-\prod_{l=1}^{G_{i}}{\bar{P}}_{C}^{H_{\ell }^{i},j}=1-{\left[P_{\text{in}}^{i}+P_{\text{a}}^{i}{\left(1-P_{\text{a}}^{j}P_{d_{ij}}\right)}^{G_{j}}\right]}^{G_{i}} \end{array}$$

Since in a typical SG scenario it is reasonable to assume statistically the NANs having similar traffic and communication characteristics, the collision probabilities between two NANs are symmetric, *i.e.*, $P_{\text{C}}^{ij}=P_{\text{C}}^{ji}=P_{\text{C}}$. Hence the overall collision probability $P_{\text{C}}^{O}$, *i.e.*, the probability that the transmissions of at least one NAN collide with the transmissions of at least one of the remaining (K−1) NANs, can be calculated as follows:

(3) PCO=▵1−P¯CK2=1−P¯CK(K−1)2

where ${\bar{P}}_{\text{C}}={\bar{P}}_{\text{C}}^{ij}$ is given in Equation (2) and the binomial coefficient K2 is due to the symmetry of the collision probabilities ${\left\{P_{\text{C}}^{ij}\right\}}_{i,j\in \{1,\ldots K\}}$. By substituting ${\bar{P}}_{\text{C}}$ in Equation (3), one has:

(4) $$\begin{array}{c} P_{\text{C}}^{O}=1-{\left[P_{\text{in}}+P_{\text{a}}{\left(1-P_{\text{a}}P_{d}\right)}^{G}\right]}^{\frac{GK(K-1)}{2}} \end{array}$$

As mentioned in the introduction, $P_{C}^{O}$ has to be under a threshold, depending on the considered application, to assure a certain level of protection on the NANs transmissions, *i.e.*,

(5) $$P_{C}^{O}\leq T_{C}$$

With $T_{\text{C}}$ denoting the largest value of the overall collision probability tolerated by the SG scenario. In the following we refer to $T_{\text{C}}$ as collision constraint. Satisfying Equation (5) is equivalent to satisfy:

(6) $$1-{\left[P_{\text{in}}+P_{\text{a}}{\left(1-P_{\text{a}}P_{d}\right)}^{G}\right]}^{\frac{GK(K-1)}{2}}\leq T_{\text{C}}$$

The largest value of *K* satisfying Equation (6), say the optimal value $K_{\text{opt}}$, is the maximum number of NANs that can be deployed in the region of area A satisfying the collision constraint $T_{\text{C}}$. Specifically, by applying the logarithm function with basis $P_{\text{in}}+P_{\text{a}}{\left(1-P_{\text{a}}P_{d}\right)}^{G}$ to Equation (6) and by recognizing that this logarithm is a decreasing function of its argument since $P_{\text{in}}+P_{\text{a}}{\left(1-P_{\text{a}}P_{d}\right)}^{G}\leq 1$, one has:

(7) $$P_{\text{C}}^{O}\leq T_{\text{C}}\Leftrightarrow K\left(K-1\right)\leq \frac{2}{G}\log_{P_{\text{in}}+P_{\text{a}}{\left(1-P_{\text{a}}P_{d}\right)}^{G}}\left(1-T_{\text{C}}\right)$$

Hence, $K_{opt}$ is obtained as the largest integer less than or equal to the maximum value satisfying Equation (7):

(8) $$K_{\text{opt}}=\left\lfloor \frac{1}{2}\left(-1+\sqrt{1+\frac{8}{G}\log_{P_{\text{in}}+P_{\text{a}}{\left(1-P_{\text{a}}P_{d}\right)}^{G}}\left(1-T_{\text{C}}\right)}\right)\right\rfloor$$

With ⌊·⌋ denoting the floor operator. It’s easy to verify that $1+\frac{8}{G}\log_{P_{\text{in}}+P_{\text{a}}{\left(1-P_{\text{a}}P_{d}\right)}^{G}}\left(1-T_{\text{C}}\right)\geq 1$, *i.e.*, $K_{\text{opt}}$ is positive. From Equation (8) it results that, for a given value of $T_{\text{C}}$, $K_{\text{opt}}$ decreases as *G* increases. The same consideration holds if $P_{\text{a}}$ increases.

In the hypothesis of NANs uniformly distributed in the region of area A and $R_{\text{TX}}<<\sqrt{A}$ (We assumed a bi-dimensional squared network region. This hypothesis is not restrictive. In fact, if the network region has dimensions *a* and *b*, respectively, one has to compare the transmission range with the smallest dimension, *i.e.*: $R_{TX}<<\min\left\{a,b\right\}$), $P_{d}=P\left(d\left(t\right)<R_{\text{TX}}\right)$ can be evaluated as: $P_{d}≃\frac{\pi R_{\text{TX}}^{2}}{A}$. By substituting this in Equation (6), the optimal NAN coverage $R_{\text{TX}}^{\text{opt}}$, *i.e.*, the maximum value of the transmission range that satisfies the collision constraint $T_{\text{C}}$, is obtained by solving Equation (6) with respect to $R_{\text{TX}}$ and by taking the maximum value:

(9) $$R_{\text{TX}}^{\text{opt}}=\sqrt{\frac{A}{\pi P_{\text{a}}}}\sqrt{1-\sqrt[{G}]{\frac{\sqrt[{\frac{GK(K-1)}{2}}]{\left(1-T_{\text{C}}\right)}-P_{\text{in}}}{P_{\text{a}}}}}$$

From Equation (9) it results that the optimal coverage increases as the SG traffic demand $P_{a}$ decreases, as expected.

In Table 1 we summarized the notation used for the parameters involved in the analysis along with their meaning, to improve the clarity of the manuscript.

## 4. Numerical Results

**Figure 2:**
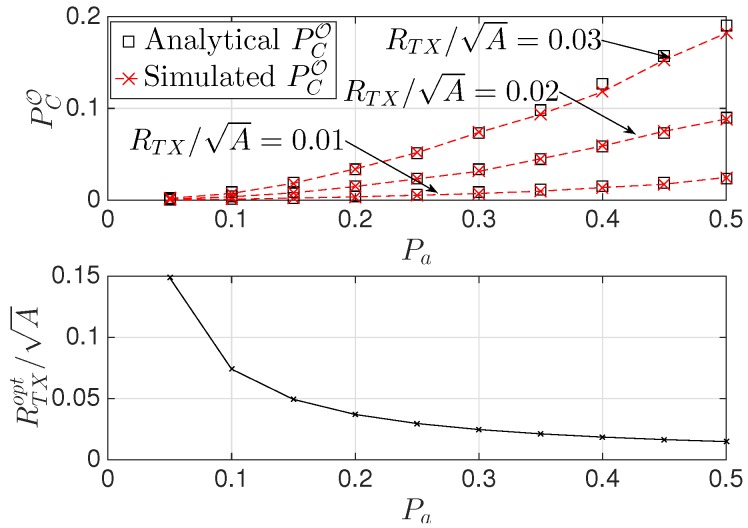
$P_{\text{C}}^{O}$ versus $P_{\text{a}}$, for different values of $R_{\text{TX}}/\sqrt{A}$.

**Figure 3:**
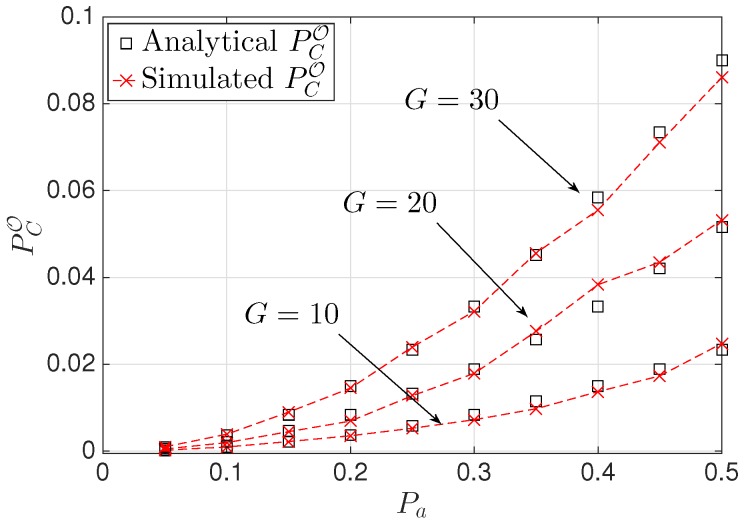
$P_{\text{C}}^{O}$ versus $P_{\text{a}}$, for different values of *G*.

Here, we validate the theoretical results through Monte Carlo simulations. We consider K=3 NANs placed randomly in a squared region of area A. Figure 2 shows the overall collision probability $P_{\text{C}}^{O}$ as a function of the SG traffic demand $P_{\text{a}}$, for three different values of the normalized transmission range, *i.e.*, $R_{\text{TX}}/\sqrt{A}=\left\{0.01,0.02,0.03\right\}$. First, we note that the theoretical results match very well the simulation results. Then, when $P_{\text{a}}$ decreases, $P_{\text{C}}^{O}$ decreases as well, since the probability of an HAN gateway being active decreases. Moreover, when $R_{\text{TX}}/\sqrt{A}$ increases $P_{\text{C}}^{O}$ increases, since it is more likely that two HAN gateways are within a distance smaller than their transmission range. In the same figure, we also plot the optimal transmission range $R_{\text{TX}}^{\text{opt}}/\sqrt{A}$ given in Equation (9) as function of $P_{\text{a}}$ for a value of the collision threshold equal to $T_{\text{C}}=0.05$. By comparison with the previous plot, we note that the theoretical results are once again confirmed. In fact, only if $R_{\text{TX}}\leq R_{\text{TX}}^{\text{opt}}$, the collision constraint is satisfied. Figure 3 shows $P_{\text{C}}^{O}$ as a function of $P_{\text{a}}$, for three different values of the number *G* of HAN gateways, *i.e.*, G={10,20,30}. Again, the theoretical results match very well the simulation results. Moreover, when *G* increases, $P_{\text{C}}^{O}$ increases as well. This result is reasonable, since it is more likely to find active HAN gateways.

## 5. Conclusions

We developed a theoretical framework for the optimal deployment of multiple closely-located NANs over TVWS spectrum. Specifically, we derived through closed-form expressions the optimal values for both the number of NANs and their coverage, *i.e.*, the maximum values assuring the given collision constraint. Such closed-form expressions reveled the highly non-linear relationship among the involved parameters as the number of NANs, their traffic demand, the number of HAN gateways and their transmission ranges. The developed analysis is crucial and preliminary for designing any effective coexistence protocol. Such an issue will be addressed in a future work along with optimization strategies for the DAU deployment.

## Figures and Tables

**Table 1:** Adopted notation.

Symbol	Definition
*K*	number of NANs deployed in the network region
A	Area of the network region
$H_{\ell }^{i}$	The *ℓ*-th HAN gateway belonging to the *i*-th NAN
$H_{m}^{j}$	The *m*-th HAN gateway belonging to the *j*-th NAN
$P_{C}^{ij}$	Collision probability of the transmissions of NANs *i* and *j*
$P_{C}^{O}$	Overall collision probability
$P_{a}$	Probability of an arbitrary HAN gateway belonging to the *i*-th NAN being active
$P_{\text{in}}$	Probability of an arbitrary HAN gateway belonging to the *i*-th NAN being inactive
*G*	Number of HAN gateways
$R_{\text{TX}}$	Transmission range of an HAN gateway
$P_{d}$	Probability of the distance d(t) between two arbitrary HAN gateways belonging
	to two different NANs being smaller than $R_{\text{TX}}$
$T_{C}$	Collision constraint, *i.e.*, the largest value of the overall collision probability tolerated by the SG scenario
$R_{\text{TX}}^{\text{opt}}$	Optimal NAN coverage, *i.e.*, the maximum value of $R_{\text{TX}}$ satisfying the collision constraint $T_{\text{C}}$
$K_{\text{opt}}$	The largest value of *K* satisfying the collision constraint $T_{C}$
